# Acquisition and adaptation of the airway microbiota in the early life of cystic fibrosis patients

**DOI:** 10.1186/s40348-016-0067-1

**Published:** 2017-01-17

**Authors:** Sébastien Boutin, Alexander H. Dalpke

**Affiliations:** 1 Department of Infectious Disease, Medical Microbiology and Hygiene, University Hospital Heidelberg, Im Neuenheimer Feld 324, 69120 Heidelberg, Germany; 2 Translational Lung Research Center Heidelberg (TLRC), Member of the German Center for Lung Research (DZL), Heidelberg, Germany

**Keywords:** Cystic fibrosis, Airways, Microbiota, Ecology, Adaptation

## Abstract

Cystic fibrosis (CF) is a genetic disease in which bacterial infections of the airways play a major role in the long-term clinical outcome. In recent years, a number of next-generation sequencing (NGS)-based studies aimed at deciphering the structure and composition of the airways’ microbiota. It was shown that the nasal cavity of CF patients displays dysbiosis early in life indicating a failure in the first establishment of a healthy microbiota. In contrast, within the conducting and lower airways, the establishment occurs normally first, but is sensitive to future dysbiosis including chronic infections with classical pathogens in later life. The objective of this mini-review is to give an update on the current knowledge about the development of the microbiota in the early life of CF patients. Microbial acquisition in the human airways can be described by the island model: Microbes found in the lower airways of CF patients represent “islands” that are at first populated from the upper airways reflecting the “mainland.” Colonization can be modeled following the neutral theory in which the most abundant bacteria in the mainland are also frequently found in the lower airways initially. At later times, however, the colonization process of the lower airways segregates by active selection of specific microbes. Future research should focus on those processes of microbial and host interactions to understand how microbial communities are shaped on short- and long-term scales. We point out what therapeutic consequences arise from the microbiome data obtained within ecological framework models.

## Introduction

Cystic fibrosis (CF) is a life-limiting autosomal recessive disorder. The cause of the disorder is a genetic mutation targeting the cystic fibrosis transmembrane conductance regulator (CFTR) gene. The most common mutation is Phe508Del (F508Del) but other mutations also modify the production or function of this ion channel [1]. The defect in the regulation of ion transport homeostasis in epithelial cells leads to a malfunction of many organs including the pancreas, the liver, the intestine, and mostly the lungs. In the airways, the genetic defect impairs mucociliary clearance as well as antimicrobial defense creating a perfect niche for microbial colonization. In turn, microbial infections, encompassing viruses, fungi, and bacteria, contribute to the elevated mortality rate in CF patients. Chronic infections by bacterial pathogens trigger airway inflammation and structural lung damage, beginning in the early life and influencing the later stage of the disease [2]. From the clinical viewpoint, improved antibiotic strategies help to increase life expectancy of CF patients [1].

For decades, microbiology of CF airways was studied by culture-based methods resulting in the main concept that infections in CF were mostly mono-specific. The prevalence of the different causative pathogens seemed to be age stratified with *Haemophilus influenzae* or *Staphylococcus aureus* being considered as pathogenic agents in the early life and *Pseudomonas aeruginosa* or *Burkholderia cepacia* complex (Bcc) being the major opportunistic pathogens during adulthood [3]. However, *P. aeruginosa* was also observed early in the life of the patients [4]. Cultured-based methods also detected species initially classified as “atypical pathogens,” mainly gram-negative bacilli, including *Stenotrophomonas maltophilia* and *Achromobacter xylosoxidans* as well as non-tuberculous mycobacteria, anaerobes and fungi. While more and more studies emerge on those pathogen, their influence on CF progression is unclear and the previously clear picture of CF mono-infections is obscured [5–8].

With technological advances in molecular biology, the microbiological view of CF shifted towards a poly-microbial concept when next-generation sequencing unraveled a high diversity of organisms living in the lower airways even in healthy persons thus contrasting the prevailing idea of a sterile lung environment [9–14]. Nowadays, it is accepted that a true microbiota in the lower airway exists and is composed to considerable parts of anaerobic bacteria [15–17]. The objective of this review will be to introduce ecological models of microbiota acquisition in the lung, give a short overview on the microbiota in the three most common sampling sites (nose, throat, and lung), and discuss the new hypothesis of a gut-lung axis connections in the early life of CF patients.

## Review

### Island model and neutral theory as ecological framework of CF microbiota development

The ecological context of infections is often neglected; yet to understand the establishment of infections the origin of pathogens has to be considered. Airways have been described within the conceptual framework of the island model where the microbiota-rich upper airways are the mainland serving as source for migrants that will colonize the microbiota-free or microbiota-poor lower airways [15, 18, 19]. Description of the lower airways as islands also indicates that different regions might undergo differential migration or selection. In CF, as well as in healthy patients, studies analyzing simultaneously the different compartments of the airways showed that the throat microbiota and most likely the oral cavity is the main source of migrants colonizing the lower airways through a route of micro-aspiration [10, 14, 20]. For the evolutionary process involved, it was shown that the neutral theory applies fairly well in healthy people as most of the bacteria in the lungs can be predicted based on a neutral model with upper airways as a source of origin [21–23]: The more abundant bacteria in the original niche will have more chances to colonize the lower airways because the balance between immigration and elimination is more decisive than regional growth selection.

### Nasal dysbiosis occurs early in life

The nasal cavity is the first filter of inhaled air which contains particulate matter and microorganisms. Small particles as well as bacteria are trapped in the mucus layer covering the nasal mucosa in the nose [24]. In healthy people the nose is known to carry commensal microbiota but also opportunistic pathogens like *S. aureus*. Roughly, a quarter of the healthy population is carrying *S. aureus* at any given time [25]. As *S. aureus* is one of the important pathogens in CF in the early life, studies aimed to evaluate the establishment of the nasal microbiota in CF and how this microbiota relates to the lower airways.

In the first months of life, a diverse bacterial community establishes in the nasal cavity. Diversity decreases during the first year but total amount of bacteria increases. This correlates with an increase in the relative abundance of *Moraxellaceae*, *Corynebacteriaceae*, and *Pasteurellaceae* in healthy people. Dominance (highest relative abundance) of those bacteria goes along with a decrease in *Staphylococcaceae* [26]. In healthy babies, it was shown by Biesbroek et al*.* that a high prevalence of *Moraxella*, *Corynebacterium*, and *Dolosigranulum* in the first year of life is associated with a more stable nasal microbiome and lower rates of respiratory infections in the consecutive periods of life [27]. This study also elegantly showed that despite differences in the composition of the nasal microbiota during the initial, very first colonization, the majority of the population finally converts to a *Moraxella*-dominated microbiota even when *Staphylococcus* was the primary colonizer. This observation indicates the first colonization is a stochastic process, depending on differences in the environment, yet intrinsic factors lead to convergence to a “healthy” nasal microbiome. In healthy adults, each person showed a unique stable microbial fingerprint but it was shown that discrete microbial types can be distinguished based on their dominant genus: *Propionibacterium*, *Moraxella*, *Corynebacterium*, or *Staphylococcus* [28, 29]. Those findings also demonstrate that in the majority of cases, in a healthy environment, *Staphylococcus* is not able to outcompete other commensals and normally does not become the dominant species.

In CF, two studies compared control to CF children in the first year of their life [26, 30]. Both studies observed a clear difference in the structure of the microbial community between CF patients and healthy controls: This shift was characterized by a decrease in *Moraxella*, *Haemophilus*, and *Corynebacterium* in CF patients, negatively correlated in the both studies with an increase in *S. aureus* abundance. Interestingly, no change in the diversity (richness and evenness of the species) of the nasal microbiota was observed when compared to healthy controls indicating that the higher abundance of *S. aureus* did not impact strongly the richness of the community.

Based on those studies, we hypothesize that the divergence between CF and healthy is not based primarily on microbial competition or microbes-microbes interaction. The divergence is most likely explained by the altered microenvironment in the nasal cavity as the CFTR mutations leads to a modified mucus composition and structure as well as defective immune response. The mucus blanket apparently favors the colonization by *Staphylococcaceae* and especially *S. aureus* while decreasing the capabilities for growing of normal, perhaps even beneficial commensals. In turn, it should be important to study in more detail the mechanisms by which the altered microenvironment shapes a different selection of the early-life nasal microbiome in CF patients.

### Seed bacteria from the nose?

Hypothesizing that nasal cavities might be a reservoir for lower airways pathogen [31–33], a dysbiosis in this niche can lead to an increase of colonization by opportunistic pathogens in the lower airways and might explain why *S. aureus* is one of the major pathogens in the early life of CF. Increased carriage of *S. aureus* in the nose might increase the chance for a first infection by *S. aureus* in lower airways if the ecological theory of island biogeography applies. Increased frequency of *S. aureus* (or other bacteria) in the nasal niche (“mainland”) will increase the probability of colonizing the lower airways (“islands”). In turn, early-life experience with facultative pathogens could prime the airways for secondary infections (e.g., by *P. aeruginosa* in the later course of the disease), perhaps by initiating and manipulating host immune reactions [31, 34–36]. Indeed, long-term changes in innate immune reactivities (“inducible innate immunity”, priming) have been shown to occur in the airways. Increased, repetitive stimulation by an altered nasal microbiota might modulate the susceptibility of lower airways towards secondary infections. It was also demonstrated that some strains found in the nose were also found in a newly transplanted lung in CF [37] arguing for communication between these niches. An altered nose microbiota could also have a distal effect by producing metabolites that may be transported to the lower airways [38–41]. Many studies have shown that *S. aureus* can enhance the growth and virulence of *P. aeruginosa*, and therefore, it can be hypothesized that *S. aureus* from the nasal cavity or from first lung infection will help *P. aeruginosa* to install [42–48]. In the same line, other nasal microbiota might exert secondary or long-term effects that modulate the lower airways’ microbiome.

Interestingly, a small part of the children with CF had a “healthy” *Moraxella*-dominated microbiome in the nose [10, 30]. It will be interesting to study whether this subgroup shows better stability of the nasal microbiome and has a better long-term clinical outcome.

### The oral cavity and the throat drive early lower airways’ microbiota

As the mucus blanket is transported through the nasal passage towards the oropharynx, it was hypothesized that oropharyngeal and nose microbiota could share similarities. However, studies in healthy as well as in CF patients showed that nasal microbiota are quite divergent from the throat microbiota [10, 20]. Definitively, the throat microbiota is more influenced by the rich, diverse, and very dense oral microbiome [49].

During the first 2 years of life, the diversity of the throat microbiota increases in the same timeline as the gut microbiome is establishing [14]. However, from 2 years on, richness and diversity then seem to be inversely correlated with age in CF indicating an instable state of the microbiome that can be correlated with the increase of the severity of the disease as well as the accumulative effects of antibiotics [3, 50]. The throat microbiota in CF is dominated by few genera including *Streptococcus*, *Veillonella*, and *Prevotella* which are also dominant genera in healthy adults [10, 14, 20, 49]. Unfortunately, to our knowledge, no studies so far tried to directly compare throat swabs or oropharyngeal samples between CF and healthy patients, especially not in young children. However, based on descriptive publications in both individual adult cohorts, it can be assumed that no major changes exist between the two cohorts in the early stage as they both exhibit a microbiota dominated by the same genera in adulthood.

One of the most dominant genera in the throat is *Streptococcus* (yet limitations in next-generation sequencing (NGS) do not necessarily allow differentiation to a precise species) and it has been shown that some Streptococci like *Streptococcus salivarius* can inhibit the growth of gram-negative bacteria [51]. Thus, microbe-microbe interactions might be an important ecological mechanism in this niche. Suppressive actions of some dominant species might explain why later on, in CF patients suffering from lower airways infection with *P. aeruginosa* or other gram-negative pathogens, these specific pathogens are found only at low abundance in the throat. A recent study from Whiley et al*.* showed that the relationship between Streptococci and *P. aeruginosa* was highly dependent on the sequence of colonization and the environmental factors: *Streptococcus* could inhibit *P. aeruginosa* only when it was the first colonizer [52]. During the first months of life, in CF patients, *Streptococcus* establishes as a dominant species [14] in the throat and therefore probably regulates the chances of infection by gram-negative bacteria in the throat. It is still unknown whether such a postulated protective effect occurs in CF patients but it is certain that it does not extend to the lower airways as gram-negative pathogens highly dominate the lung microbiome during the later stage of the disease [1]. Microbial interactions within the specific niche of the throat might result in a quite stable microbiota that is more influenced by the oral cavity and less prone to alterations even in CF patients.

### The lung microbiota: a dedicated balance of migration and elimination

**Fig. 1:**
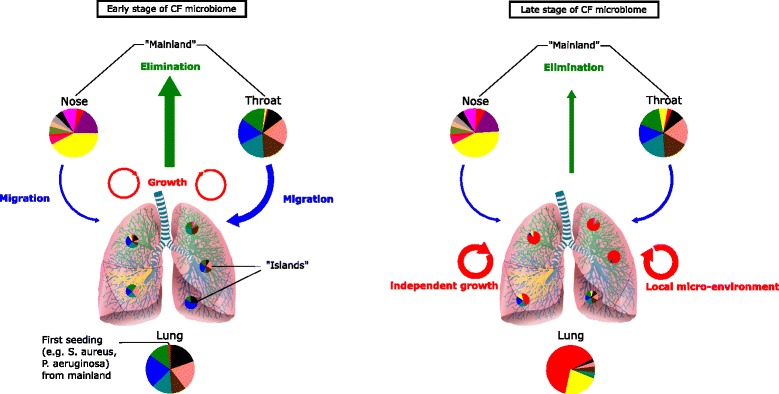
Theoretical acquisition and evolution of the microbiome in CF airways. In the early stage of CF, migration of bacteria from the nose and throat (considered as the mainland) will seed the lower airways. The lung microbiome in the early phase of CF resembles the one from the throat. Thus, migration, growth, and elimination of the microbiota with the throat as main source also initially is balanced as observed in healthy people. With aging, changes in the regional conditions (mucus, nutrients, pH, clearance, and immunity) lead to an unbalance in the equilibrium between migration and elimination thus favoring regional growth in the lower airways of typical or atypical CF pathogens. This overgrowth probably is not linked to major changes in the source niches (nose and throat), yet pathogens may originate from there. Microenvironment changes trigger the segregation of the lung’s microbiome from the throat’s microbiome creating a third independent microbiome in the airways

The presence of a true commensal microbiota establishing in the lower airways is still a matter of debate especially because lung microbiota resembles more a transient colonization from the upper airways than a true stable growth of commensals [15]. Many studies using NGS showed that the throat microbiota is closely related to the lung microbiota, the latter sampled by bronchoalveolar lavage (BAL) or expectorated sputum in healthy and CF patients [10, 20, 53]. Proximity and interconnection of both compartments argues for micro-aspiration as the main process leading to bacterial immigration from the upper to the lower airways. As a consequence, the first microbiota acquired in early life probably originates from migrants from the throat microbiota that are able to colonize and reproduce in the lower airways [10, 18–20, 54] (Fig. 1). Indeed, it was shown that in young children with CF, the lower airways’ microbiota resembled the one in the throat and this also holds true for adults unless chronic infections with specific pathogens (e.g., *P. aeruginosa*, *Burkholderia*) are establishing [10]. Work of our own also shows that repeated sputum samples of CF patients are not overly stable when analyzed at the individual genus distribution, yet the overall composition matched well a corresponding throat sample. Data argue for repeated cycles of microaspiration and removal of bacteria in the lower airways of the CF patients in early life with the throat being the source.

As a proof of concept, in less severe disease, no classical pathogens are observed by NGS or by culture and the same genera are dominating the lower airways’ microbiome and the oropharyngeal community. *Streptococcus*, *Prevotella*, *Veillonella*, and *Neisseria* are the most common and abundant genera in the lower airways in young CF patients as well as in healthy people [17, 20, 22, 53, 55]. A high intra-individual variation in the structure of the microbiome was highlighted in different studies, mostly because the lung is a heterogeneous niche and does not possess a homogeneous microbiome [56–59]. Individual patients exhibited a personalized microbiome in the lower airways. Confirming these results, sections of lungs taken during transplantation possessed a unique microbiome in CF disease [56, 57]. This spatial heterogeneity slightly contrasts results obtained in healthy patients who show a quite good homogeneity in the different lobes of the lungs. This is probably because the microbiome in the healthy lung is the result of microbial immigration and elimination while in diseased patients, effects of local growth might be more determinant [19]. Renwick et al*.* also showed that, despite a close relationship regarding the dominant taxa, BAL microbiota from young CF patients had significant differences from healthy controls [60]. Those differences between healthy and CF subjects mostly rely on the global diversity which decreases in CF. The results argue for the development of an early dysbiosis in young CF patients that starts from colonization from upper airways but then gets independent and develops separately (Fig. 1).

The contrasting results between the studies using transplanted lungs [56, 57] and BAL/sputum [10, 60] in CF compared to throat microbiota might be explained by the status of the disease. CF lungs from very young patients exhibit an overlapping microbiome to the throat. However, in advanced stage disease, when a pathogen overgrows, the throat microbiota is not a good surrogate anymore of the lower airways [10, 20]. Some studies observed that throat microbiota did not reflect the microbiota of the lower airways in some CF patients [10, 56, 61]. All those studies were analyzing patients with chronic infections in the lower airways showing a high dominance of known classical pathogens. These results indicate that chronic infections are an important trigger in the shift in the microbiome of the lower airways. Mostly, known pathogens like *P. aeruginosa*, *S. aureus*, *Bordetella*, *Haemophilus*, and *Burkholderia* reach a high dominance through different modes of selection and high dominance is mostly found only in an adult or aged cohort. The same pattern is likely to occur with the emerging atypical CF pathogens like *S. maltophilia* and *A. xylosoxidans*, non-tuberculous mycobacteria, and fungi [5]. High dominance of one species correlates with a decrease in diversity, anaerobe load, and lung function [10, 55, 62].

It can thus be hypothesized that the neutral model holds true only at the beginning of airway colonization in CF (Fig. 1): The more abundant genera in the throat are the more frequent colonizers in the lower airways. In contrast to healthy people, in later stage CF with progressing disease, microbial communities are clearly under selection. The balance between migration and elimination is disturbed and/or regional growth selection processes overwhelm the fine-tuned balance. Only then, some pathogens adapt and grow in the lower airways as evidenced by their higher abundancy in the lower airways as compared to the source (nose or throat) [21]. This change represents a segregated individual development that probably depends more on microenvironmental factors than on the bacteria themselves because the spectrum of invading migrants is not overly different. In turn, it must be concluded that the local environment factors (pH, nutrient and mucus composition, clearance processes, and immunity) differ. It might also be that those differences not directly select for growth of pathogens but affect the interplay in the immigrating community (e.g., anaerobes-pathogen interactions), thus favoring outgrowth of certain bacteria and reducing the diversity of colonizers.

### Decreasing microbial diversity in later stage CF disease

The primary microbial colonization seems to be unchanged in CF as migrants come from a similar pool of species: overlap with throat community, for which minor differences between healthy and diseased patients exist, is observed. However, microbial clearance and regional growth are undoubtly changed by the CFTR mutation itself. Deficiency in mucociliary clearance and immune response will modify the capacity to manage the bacterial load in the lower airways while the modification of the mucus composition itself will alter growth conditions. Mucus in healthy people is a thin layer of low-nutrient environment which is highly dynamic while in CF, the mucus layer is thicker with global changes in osmolarity, oxygen concentration, and decreased mobility [63]. Those differences could in theory favor a different microbiome establishment, possibly allowing the dominance of fewer bacteria (*P. aeruginosa, H. influenzae, S. aureus, Burkholderia cepacia* complex), thus explaining the decrease in diversity observed in CF compared to healthy lung with aging [3, 50, 63]. Thus, environment-driven selection processes will shape a different microbiome despite similar processes of acquisition [64]. An increasingly important factor could also be the frequent usage of antibiotics, but due to the complexity and variability of therapeutic regimens, this will be difficult to control in microbiota studies. Antibiotics with a broad spectrum are potentially a strong selection pressure that will affect more the whole microbiota than the targeted pathogens. Furthermore, there is evidence that the success of antimicrobial therapy is impacted by microbe-microbe interactions, indicating that a personalized therapy which takes into account the individual microbiome should be favored [65].

### What is the role of anaerobes in CF?

In the last years, many studies observed a negative correlation between gram-negative pathogens and commensal anaerobes, notably *Prevotella* and *Veillonella* [3, 10, 66]. The reduction of the anaerobe load was linked to a global worsening of the disease. This went along with a decrease of the clearance index and increased inflammation even in the absence of *P. aeruginosa* [55] or other pathogens. It was hypothesized that commensal anaerobes in the lower airways exert a protective role [67, 68]. Contrasting studies showed that anaerobes are linked to exacerbation and might create a favorable niche for recognized CF pathogens [67, 69, 70]. Furthermore, antibiotic use in CF drives a strong selection for antibiotic-resistant bacteria from the whole microbiota. Therefore, an acquired resistance from residential commensals can also spread via passive resistance to CF pathogens [68, 71]. Those contradictory observations lead to the question whether anaerobes in CF should be covered by antibiotic regimens. Of note, many NGS-based studies observed a negative impact of reduced anaerobe abundances in patient’s health. Definitively, more studies in the early steps of the disease and the relationship anaerobes-pathogens and anaerobes-health will be needed [67]. Those studies probably will have to use metagenomic, metatranscriptomic, or proteomic approaches to allow functional analysis instead of mere description of the microbiota composition [72, 73].

### A new connection: the gut-lung axis

While most of the studies focused on lung in CF, a new focus is emerging which is the gut microbiome. The gut microbiome is intensively studied in humans and has been linked to several diseases and phenotypes. The importance of this bacterial community especially relates to the maturation of the immune system. It was observed that the neonatal period is highly important to establish a mature immune system. CFTR mutations affect the gut and airway micro-environment inducing modifications in the colonization process of microorganisms even in the absence of antibiotics [3]. Patients suffering from CF present with intestinal dysfunction, pancreatic defect, and thicker mucus in the intestinal lumen that will affect the structure and function of this ecological niche [74–77]. It is well characterized that the establishment of a beneficial community in the gut is associated with systemic health and immunity at distal sites [78]. One of those distal sites is the lung which is the most important niche under constant microbial threat in CF. The influence of the gut microbiome on lung health has been termed the gut-lung axis. This theory relies on the fact that some microbes in the gut might affect directly the lung microbiome via seeding (during oesophageal reflux) or more likely indirectly via transported metabolites through the bloodstream or by influencing the systemic immune response.

Airways’ microbiota develops in the same time as the gut microbiota, starting with colonization right after birth. In CF, there is some evidence that dysbiosis in the gut occurs in the early life of patients right after birth [79]. This dysbiosis is most likely linked to the genetic mutation as the structural and functional defects of the gut originate from it. Gut inflammation is indeed observed in young CF patients [80]. Experiments with CFTR knockout mice showed that dysbiosis occurs even in the absence of antibiotics and that the gut presents with abnormal structure [81, 82]. In CF patients, alterations of the lung microbiome also correlate to known gastrointestinal complications [76, 77]. A decrease of beneficial commensals (*Bifidobacterium* and *Clostridia*), known to help the maturation of the immune system and protecting against infection in healthy patients, was observed. The colonization by the genus *Veillonella* also seems to be altered by the CFTR mutation [83].

Early dysbiosis in the respiratory tract in CF patients [60] might be associated with dysbiosis in the gut [78]. Some genera (*Prevotella* and *Veillonella*) are known to belong to both niches, gut and lung. Following this rationale, Madan et al*.* designed a keystone longitudinal study showing that gut and lung microbiome are two distinct entities. However, the gut microbiota shared with the airways a core set of common bacteria dominated by *Streptococcus* and *Veillonella* in CF children up to 21 months [14]. More than eight genera which increased or decreased over time in the gut also showed similar changes in the respiratory tract. Moreover, seven genera colonizing the gut preceded the colonization in the lungs. Of note, respiratory bacterial diversity and microbial structure were correlated to dietary modifications (breast-feeding and introduction of solid food). The data are compatible with a role of micro-aspiration from the oral cavities in the colonization of the lower airways [14]. Studies on probiotics administration in CF children and young adult showed a beneficial effect on the lung phenotype by decreasing the frequency of pulmonary exacerbation and restoration of gut microbiota [84, 85]. Intriguingly, those findings allow for speculation that dietary changes and probiotics might be used to manipulate also the respiratory microbiota but whether this will be possible only during a narrow window of first post-natal colonization or might even work in elder people is unclear.

## Conclusions

Acquisition of the airways’ microbiota occurs in the first days of life in CF patients as well as in healthy patients. The same process of colonization is observed in all compartments of the upper airways with a first colonization by a rich pool of bacteria that rapidly adapts during the first months of life and is selected based on the capability to grow and resist clearance. The gut microbiome is interconnected to the early respiratory microbiome and might play a role as initial source. The oropharyngeal community of young CF patients shows few differences compared to healthy controls while the nasal cavity shows marked differences that establish early in life. For clinical purposes, it could be valuable to restore a normal nasal microbiome thus reducing the *S. aureus* carriage early in the life of the patients. In the lungs, limitations in sampling preclude longitudinal studies to elucidate the acquisition of microbiome in the first days of life. However, studies indicate that the throat microbiota well reflects the lower airways in young CF children. Establishment of the lower airways’ microbiome is strongly impacted by the upper airways’ bacterial community and starts as suggested by an island ecology model. Neutral model applies for healthy lung microbiome, yet in CF, microenvironment factors contribute over time to the establishment of a bona fide local microbial community that segregates from the influence of the upper airways “mainland”.

### Open questions and therapeutic implications

From the reported findings, a number of questions arise which should be subject of future research: Is there an influence of the gut microbiome on the respiratory microbiome extending over the early time period of establishment? What is the functional role of the anaerobes found in the lower airways of CF patients? Do they just mirror the source of colonization from upper compartments or do they play a functional role of its own (disease aggravating or even beneficial)? What are the interactions of classical pathogenic bacteria with commensals and how do these interactions evolve to allow establishment of dominant infections at later disease stage? To what extent contributes the disturbed nasal microbiome to an environment favoring infections of the lower airways? What are the local host factors (microenvironment, immunity) that allow selection processes of local bacteria growth to occur in later microbiome development? What host factors differ in CF patients resulting in deviation from the neutral ecology model and resulting in a bona fide lung microbiota? Therapeutic implications could be: Can nutritional changes be used to modify the respiratory microbiome? Could narrow-spectrum antibiotics or a targeted antibiotic strategy be useful to correct environment changes that evolve over time in the lower airways? Might manipulation/correction of the disturbed early life nasal microbiome be a strategy to influence later lower airway microbiome composition (e.g., local *S. aureus* therapy)? Might commensal “probiotics” be used to correct the diseased CF microbiome?

Finally, most of the NGS-based studies so far focus on taxonomical structure or composition. However, in order to better understand relationships among the different bacterial communities in the airways as well as to elucidate the functional mechanisms leading to dysbiosis, true metagenomic, transcriptomic, and proteomic studies targeting the whole genome, transcriptome, and proteome will be necessary. Definitively, NGS studies bear the potential to further increase our understanding of the disturbed CF microbiota.

## Abbreviations

Bcc: *Burkholderia cepacia* complex
CF: Cystic fibrosis
CFTR: Cystic fibrosis transmembrane conductance regulator
NGS: Next-generation sequencing
